# Supplementary material for: Asynchronous magnetic resonance elastography: Shear wave speed reconstruction using noise correlation of incoherent waves
Source: Magn Reson Med. 2022 Oct 27;89(3):990–1001. doi: 10.1002/mrm.29502 (PMC9792433; doi:10.1002/mrm.29502)
Supplement: Supplementary file 1 — DATA S1 MATLAB code used to generate the numerical simulations of Figure 1 [file MRM-89-990-s002.zip › k-Wave/helpfiles/toneBurst.html]

toneBurst :: Functions (k-Wave)


# toneBurst

Create an enveloped single frequency tone burst.

## Syntax

```
signal = toneBurst(sample_freq, signal_freq, num_cycles)
signal = toneBurst(sample_freq, signal_freq, num_cycles, ...)
```

## Description

`toneBurst` creates an enveloped single frequency tone burst for use in ultrasound simulations. If an array is given for the optional input `'SignalOffset'`, a matrix of tone bursts is created where each row corresponds to a tone burst for each value of the `'SignalOffset'`. If a value for the optional input `'SignalLength'` is given, the tone burst/s are zero padded to this length (in samples). Several examples are given below.

```
% return and plot a 1 MHz tone burst with 10 cycles and a Gaussian envelope sampled at 20 MHz
toneBurst(20e6, 1e6, 10, 'Plot', true);
```

```
% specify several signals with different temporal offsets
sample_freq          = 20e6;
signal_freq          = 1e6;
num_cycles     	     = 10;
num_ring_up_cycles   = 2;
num_ring_down_cycles = 2;
signal_offsets       = [0, 20, 40];

% create signals
signals = toneBurst(sample_freq, signal_freq, num_cycles, 'Envelope', [num_ring_up_cycles, num_ring_down_cycles], 'SignalOffset', signal_offsets); 

% plot signals
figure;
t_axis = (1:size(signals, 2)) / sample_freq;
stackedPlot(1e6 * t_axis, signals);
xlabel('Time [\mus]');
ylabel('Signal Number');
```

## Inputs

|  |  |
| --- | --- |
| `sample_freq` | sampling frequency [Hz] |
| `signal_freq` | signal frequency [Hz] |
| `num_cycles` | number of sinusoidal oscillations |

## Optional Inputs

Optional 'string', value pairs that may be used to modify the default computational settings.

| Input | Valid Settings | Default | Description |
| --- | --- | --- | --- |
| `'Envelope'` | `'Gaussian'` `'Rectangular'` `[num_ring_up_cycles, num_ring_down_cycles]` | `'Gaussian'` | Envelope used to taper the tone burst. The last option generates a continuous wave signal with a cosine taper of the specified length at the beginning and end. |
| `'Plot'` | *(Boolean scalar)* | `false` | Boolean controlling whether the created tone burst is plotted. |
 `'SignalLength'` | *(integer)* | `'auto'` | Signal length in number of samples. If longer than the tone burst length, the signal is appended with zeros. | `'SignalOffset'` | *(integer)* | `0` | Signal offset before the tone burst starts in number of samples. |

## Outputs

|  |  |
| --- | --- |
| `signal` | created tone burst |

## Examples

- Steering A Linear Array
- Snell's Law And Critical Angle Reflection

## See Also

`gaussian`
